# Understanding imported malaria in western Nepal: Implications for malaria control and elimination

**DOI:** 10.1371/journal.pgph.0006079

**Published:** 2026-03-27

**Authors:** Shashi Kandel, Madan Koirala, Gokarna Dahal, Rohit Kumar Sah, Prajjwal Upadhayaya, Kanlaya Jongcherdchootrakul, Phanthanee Thitichai

**Affiliations:** 1 Department of Health Services, Epidemiology and Disease Control Division, Ministry of Health and Population, Kathmandu, Nepal; 2 Save the Children, Kathmandu, Nepal; 3 Central Department of Public Health, Institute of Medicine, Tribhuvan University, Maharajgunj, Kathmandu, Nepal; 4 Department of Military and Community Medicine, Phramongkutklao College of Medicine, Thailand; 5 Department of Disease Control, Division of Epidemiology, Field Epidemiology Training Program, Ministry of Public Health, Thailand; University of Oxford, UNITED KINGDOM OF GREAT BRITAIN AND NORTHERN IRELAND

## Abstract

Imported malaria has emerged as a key challenge for malaria elimination efforts in Nepal, particularly in border regions with high labor migration to malaria-endemic areas. Identifying the geographic origin and demographic characteristics of imported cases is essential to inform targeted interventions and prevent local transmission. A community-based cross-sectional study was conducted in Narainapur Rural Municipality, Banke District, from November 2024 to January 2025. Data were collected from 113 imported malaria cases reported between October 1, 2023, and September 30, 2024, using structured face-to-face interviews and medical record reviews. Socio-demographic, travel, occupational, and clinical information were analyzed descriptively. All 113 respondents were male, with the majority aged 20–29 years (46.02%). Most belonged to the Muslim ethnic group (84.07%). Almost all (99.11%) had traveled to India for work, with Maharashtra being the primary destination state (96.47%) and South Mumbai the most common destination area (95.60%). Bhendi Bazar (52.21%), Bohri Muhalla (23.90%), and Null Bazar (7.96%) were the most frequently reported localities. The predominant occupation was catering services (71.43%). A significant number (85.84%) lacked fixed housing and stayed on the streets. Only 56.64% had received malaria-related information before travel, and over half (59.30%) were unwilling to use Long Lasting Insecticidal Nets, citing unsuitability for their living conditions. Imported malaria in Narainapur disproportionately affects young male labor migrants, predominantly from the Muslim community, whose occupational and living conditions place them at continued risk of infection. Frequent mobility, night and mixed indoor–outdoor work, and unstable housing limit the effectiveness of conventional prevention measures. The risk of malaria among Nepali labor migrants is not random but concentrated in well-defined urban hotspots in destination countries. These findings highlight the urgent need for targeted, migrant-sensitive prevention strategies and improved cross-border collaboration to support Nepal’s malaria elimination efforts.

## Introduction

Malaria is a life-threatening disease caused by protozoan parasites of the genus Plasmodium, transmitted through the bite of infected female Anopheles mosquitoes [1]. Of the more than 200 known *Plasmodium* species, five infect humans—*P. falciparum, P. vivax, P. malariae, P. ovale,* and *P. knowlesi*, the latter being a zoonotic parasite primarily found in Southeast Asia [2]. Symptoms range from fever, chills, rigor and headaches to severe complications such as seizures and coma, with the incubation period typically ranging from 7 to 30 days. Some species, like *P. vivax* and *P*. *ovale*, can cause relapses months after initial infection [3].

Malaria remains a pressing global health issue, with an estimated 263 million cases annually, an incidence of 60.4 cases per 1000 population at risk, and 597 000 deaths globally in 2023, a mortality rate of 13.7 per 100 000 [4]. While the highest burden is observed in the WHO African region, malaria continues to affect millions across Asia. The WHO South-East Asia Region (SEAR) recorded an 82.4% reduction in malaria cases between 2000 and 2023, reflecting significant progress in control efforts. India alone accounted for half of the region’s estimated malaria cases. Nepal, which borders India, reported 15 indigenous malaria cases in 2023, an encouraging milestone in its journey toward elimination [4]. However, as indigenous cases have declined, the threat of imported malaria has grown more prominent, primarily driven by labor migration to endemic regions [5]. Nepal’s open borders with India facilitates large-scale population movement, and many Nepali migrants travel to urban areas in the neighboring country for work. A significant number return with malaria infections, contributing to local transmission [6]. Between 2007 and 2013, 30–44% of Nepal’s malaria cases were imported [7,8] a figure that exceeded 95% by 2021/22 [9]. Narainapur rural municipality in Banke district has been a persistent hotspot, driven by high labor migration to endemic areas [10,11]. Despite ongoing efforts [12,13], imported cases in Narainapur have continue to rise, with over 100 cases reported in the first eight months of 2024 (unpublished report—Epidemiology and Disease Control Division).

At the community level, malaria surveillance in Narainapur is supported by Village Malaria Workers (VMWs), who are part of Nepal’s national malaria elimination program. VMWs contribute to routine malaria control activities, including identification of febrile cases, use of rapid diagnostic tests, facilitation of treatment and referral, and reporting through the health system. In border and high-migration settings such as Narainapur, their presence is particularly relevant for the early detection of imported malaria cases among returning labor migrants, thereby supporting timely public health response and prevention of secondary transmission. However, critical gaps remain in the surveillance and understanding of imported malaria. Routine surveillance system fails to capture detailed geographic information on the origin of infection, such as the specific cities, states, or localities in India, limiting the ability to design targeted cross-border interventions. Additionally, demographic details including occupation are oversimplified or broadly recorded as “migrant labor”, these data are vital for identifying high-risk populations and understanding how exposure to malaria may vary across different groups, locations and occupation. The lack of both geographic specificity and detailed demographic characteristic limits the effectiveness of prevention strategies and weakens cross-border collaboration. Hence, this study aims to identify the demographic characteristics and geographic origin of imported malaria cases in Narainapur, Banke.

## Materials and methods

### Study design and setting

A cross-sectional study was conducted to analyze imported malaria cases in Narainapur rural municipality, Banke District, Lumbini Province, Nepal. Data collection took place from November 20, 2024, to the end of January 2025. Narainapur is bordered by Raptisonari rural municipality to the north, Duduwa municipality to the west, and India to the east and south. It is divided into six wards, covering 172.34 square kilometers, with a population of 34,942. The religious composition of the population is 54.5% Hindu, 45.0% Muslim, and less than 1% Christian, Buddhist, or other religions. The study focused on imported malaria cases reported between October 1, 2023, and September 30, 2024.

### Study participants and sampling

Imported malaria refers to individuals diagnosed with malaria (positive for WHO pre-qualified malaria Rapid Diagnostic Tests (mRDT) or microscopy) who traveled to malaria-endemic regions within the past 30 days or were classified as imported cases by the national malaria elimination program. Of the 170 reported cases, 15 individuals declined to participate, and 42 could not be reached due to migrant work abroad, leaving 113 participants for the final sample.

### Data collection tool and technique

A structured questionnaire was deployed on mobile devices using KoboCollect, an open-source application designed for offline data collection in field settings, to facilitate efficient and accurate data entry. The validity and reliability of the questionnaires were rigorously assessed. Content validity was evaluated using the Content Validity Index (CVI), with a panel of three subject matter experts rating each item on a four-point Likert scale for relevance and clarity. The Item-Level CVI (I-CVI) values exceeded 0.78, while the Scale-Level CVI (S-CVI/Ave) reached 0.99 for relevance and 0.97 for clarity, indicating strong content validity [14]. Face validity was assessed through a pilot test involving 15 imported malaria cases from another province with a similar epidemiological and migration context. These participants were not included in the final study sample. Feedback from the pilot test was used to refine and improve the clarity of the questionnaire. To ensure reliability and minimize interviewer bias, three data collectors were trained prior to the commencement of data collection. Data collection progress was monitored in real time through KoboToolbox, and all data were securely synced to cloud storage for safe access and backup. Data collection methods included medical record review, where relevant clinical information was extracted from the health records of confirmed malaria cases, and structured, face-to-face interviews conducted to obtain sociodemographic, socioeconomic, and behavioral data from individuals diagnosed with imported malaria.

### Data management and analysis

Data management involved thorough cleaning to identify and address missing, incomplete, or inconsistent responses, with outliers reviewed for potential entry errors. When discrepancies were found, participants were re-contacted to verify and update the information. To ensure confidentiality, all data were anonymized and access was restricted to authorized personnel only. Categorical variables were analyzed and presented as frequencies and percentages, while continuous variables were summarized using median and interquartile range (IQR).

### Ethical considerations

Ethical approval for the study was obtained from the Nepal Health Research Council (Registration No. 526_2024) and Narainapur municipality (Rec. No. 239, 2081/082) before the commencement of data collection. The written informed consent was obtained from each respondent prior to data collection. Participants were informed that their participation was completely voluntary, that they could refuse to answer any question, and that they could stop the interview at any time.

## Results

### Socio-demographic characteristics of participants

Table 1 displays the socio-demographic characteristics of 113 imported malaria cases from Narainapur rural municipality, Banke, Nepal. The median age was 25 years and all cases were male (100%). The majority belonged to the 20–29 years age group (46.02%), followed by 30–39 years (23.89%). In terms of ethnicity, the almost all followed Islam religion (84.07%) and 76.11% were married. A substantial majority (91.15%) of participants were not formally educated.

**Table 1:** Socio-demographic distribution of imported malaria Cases, Narainapur municipality, Banke, Nepal, 2024 (n = 113).

Characteristics		Frequency (n)	Percentage (%)	95%CI
**Age in years** Median (Q1-Q3): 25 (21–33) NA NA				NA
**Age Groups**				
	0 - 9	0	0.00	0.00 - 0.00
	10 - 19	18	15.93	9.18 - 22.68
	20 - 29	52	46.02	36.83 - 55.21
	30 - 39	27	23.89	16.03 - 31.76
	40 - 49	12	10.62	4.94 - 16.30
	>=50	4	3.54	0.13 - 6.95
**Sex**				
	Male	113	100.0	100.00 - 100.00
**Ethnicity**				
	Muslim	95	84.07	77.32 - 90.82
	Janajati	12	10.62	4.94 - 16.30
	Dalit	6	5.31	1.18 - 9.44
**Religion**				
	Islam	95	84.07	77.32 - 90.82
	Hinduism	18	15.93	9.18 - 22.68
**Marital Status**				
	Married	86	76.11	68.24 - 83.97
	Never Married	26	23.01	15.25 - 30.77
	Widowed/Widower	1	0.88	-0.84 - 2.61
**Education**				
	Illiterate	64	56.64	47.50 - 65.77
	Self-educated	39	34.51	25.75 - 43.28
	Primary (1–5)	6	5.31	1.18 - 9.44
	Lower secondary (6–8)	4	3.54	0.13 - 6.95

### Travel and work-related information of imported malaria cases

**Fig 1:**
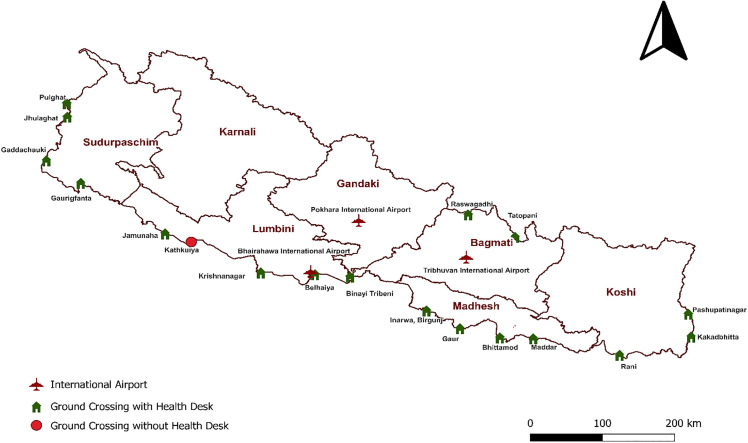
Point of Entry and Health Desk in Nepal. **(Shape file:** **https://geotechspace.com.np/useful-data**).

**Fig 2:**
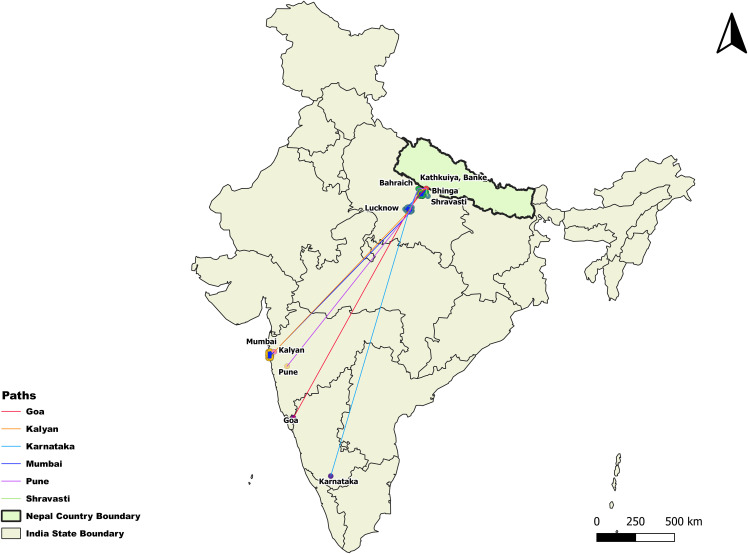
Travel Destination of Imported Malaria cases, Narainapur Banke, Nepal, 2024 (Shape file: https://www.indianremotesensing.com/2017/01/Download-India-shapefile-with-kashmir.html).

Table 2 displays the travel and work-related information of imported malaria cases. All participants (100%) had traveled to India, and all reported crossing into India through Kathkuiya, Banke, a non-designated ground crossing point where no health desk has been established, unlike other official ground crossing that are equipped with health desk (Fig 1). Among Indian states, Maharashtra was the primary destination (96.46%) with Mumbai being the most frequently visited city within Maharashtra (99.08%). Within Mumbai, Bhendi Bazar (54.63%) and Bohri Muhalla (25.00%) were the most common neighborhoods. A few individuals also reported traveling to other states such as Goa (0.88%), Karnataka (0.88%), and Uttar Pradesh (1.77%) (Fig 2).

**Table 2:** Travel and work-related information of imported Malaria Cases, Narainapur municipality, Banke, Nepal, 2024 (n = 113).

Category	Location/Detail	Frequency (n)	Percentage (%)		95% CI
**Destination Country**					
	India	113	100.00	100.00 - 100.00	
**State**					
	Maharashtra	109	96.46	93.05 - 99.87	
	Goa (North Goa / Anjuna)	1	0.88	-0.84 - 2.61	
	Karnataka (Bengaluru (Gandhinagar/Majestic)	1	0.88	-0.84 - 2.61	
	Uttar Pradesh (Shrawasti)	2	1.77	-0.66 - 4.20	
**Maharashtra**					
	Pune	1	0.92	-0.84 - 2.68	
	Mumbai	108	99.08	97.32 - 100.84	
	- Bhendi Bazar	59	54.63	45.45 - 63.81	
	- Bohri Muhalla	27	25.00	17.02 - 32.98	
	- Null Bazar	9	8.33	3.24 - 13.43	
	- Bharat Bazar	2	1.85	-0.63 - 4.34	
	- Others	11	10.19	4.61 - 15.76	
**Travel Purpose**					
	Work	112	99.10	97.36 - 100.84	
	Visit friends and families	1	0.90	-0.84 - 2.64	
**Ground Crossing Point**					
	Kathkuiya (from Nepal)	113	100.00	100.00 - 100.00	
**Travel Duration to Reach Destination (Days)**					
	Median (Q1-Q3): 3 (2–3)	NA	NA	NA	
**Migration Frequency**					
	≤ once a year	41	36.30	27.43 - 45.17	
	2–3 times a year	65	57.50	48.39 - 66.61	
	4–5 times a year	4	3.50	0.11 - 6.89	
	>5 times per year	3	2.70	-0.29 - 5.69	
**Will revisit Same Place**		98	86.70	80.44 - 92.96	
**Type of work (n = 112)**					
	Catering Services	80	71.43	63.10 - 79.76	
	Hotel and Bakery	14	12.50	6.40 - 18.60	
	Construction work	8	7.14	2.39 - 11.89	
	Factory work	3	2.68	-0.30 - 5.66	
	Not Specific	7	6.25	1.79 - 10.71	
**Work Environment (n = 112)**					
	Outdoor	9	8.04	3.02 - 13.05	
	Indoor	9	8.04	3.02 - 13.05	
	Both Outdoor and Indoor	94	83.93	77.16 - 90.70	
**Work Shift (n = 112)**					
	Day	5	4.46	0.66 - 8.27	
	Night	5	4.46	0.66 - 8.27	
	Both	102	91.07	85.81 - 96.33	
**Place to live**					
	No fix place to live (spends nights on street)	97	85.84	79.41 - 92.27	
	Pakka house^*^	8	7.08	2.35 - 11.81	
	Kaccha house^#^	6	5.31	1.18 - 9.44	
	Semi Pakka house^!^	2	1.77	-0.66 - 4.20	

^†^Catering services refer to event-based food service activities, including food preparation, serving, dishwashing, and kitchen support during functions or gatherings

*Permanent structure made with materials like brick, cement, or concrete

^#^Temporary structure made from materials like mud, thatch, or bamboo

^!^partly permanent structure made with both durable (e.g., brick) and temporary (e.g., tin, thatch) materials

The primary reason for travel was work (99.10%). The median duration of travel to reach destination was 3 days. Most individuals usually migrate 2–3 times a year (57.50%), and a high proportion (86.70%) intended to revisit the same place. Among the 112 individuals who traveled for work, the majority were involved in catering services (71.43%), followed by hotel and bakery work (12.50%). Most worked in both indoor and outdoor environments (83.93%) and had mixed day and night shifts (91.07%).

Living conditions were generally poor, with 85.84% mentioning no fixed place to stay and often sleeping on the street. Only a small proportion lived in pakka (7.08%), kaccha (5.31%), or semi-pakka (1.77%) housing structures (Table 2).

### Clinical characteristics and willingness to prevent malaria infection

Table 3 shows the clinical characteristics and willingness to prevent malaria infection. *P. vivax* was the most frequently identified species (61.95%). All cases were classified as uncomplicated malaria, with fever and chills being the most commonly reported symptoms. A majority (64.60%) returned with fever or developed symptoms within the first week of return (12.39%). While 65.49% were aware that malaria is preventable and 56.64% had received malaria-related information before travel, only 40.70% were willing to use LLINs, primarily due to inconvenience in using them. In contrast, all participants expressed willingness to use malaria chemoprophylaxis.

**Table 3:** Clinical characteristics and willingness to prevent malaria infection among imported malaria cases, Narainapur municipality, Banke, Nepal, 2024 (n = 113).

Characteristics		Frequency (n)	Percentage (%)	95% CI
***Plasmodium* Species**				
	*P. vivax*	70	61.95	52.99 – 70.90
	*P. falciparum*	30	26.55	18.41 - 34.69
	*P. mix*	13	11.50	5.62 - 17.39
**Sign and Symptoms**				
	Fever	113	100.00	100.00 - 100.00
	Chills and Rigor	108	95.58	91.78 - 99.37
	Headache/Body ache	94	83.19	76.29 - 90.08
	Nausea/Vomiting	3	2.65	-0.31 - 5.62
	Abdominal pain/Distention	0	0.00	0.00 - 0.00
**Severity Classification**				
	Uncomplicated Malaria	113	100.00	100.00 - 100.00
**Duration between return and symptom onset**				
	Returned with fever	73	64.60	55.78 - 73.42
	Fever within 1–7 days	14	12.39	6.31 - 18.46
	Fever within 8–14 days	3	2.65	-0.31 - 5.62
	Fever within 15–30 days	11	9.73	4.27 - 15.20
	Fever on 31 + days	12	10.62	4.94 - 16.30
**Knew malaria is a preventable disease**		74	65.49	56.72 - 74.25
**Received malaria information pre-travel**		64	56.64	47.50 - 65.77
**Willing to use chemoprophylaxis**		113	100.00	100.00 - 100.00
**Willing to use LLIN**		46	40.70	31.64 - 49.76
**Reason behind not willing to use LLIN**				
	Lack of Awareness	0	0.00	0.00 - 0.00
	Inconvenience in setting nets	67	100.00	100.00 - 100.00
	Cultural Practices	0	0.00	0.00 - 0.00
	Concerns about heat or discomfort	0	0.00	0.00 - 0.00
	Low Perceived Risk	0	0.00	0.00 - 0.00
	Reliance on other prevention methods	0	0.00	0.00 - 0.00

## Discussion

The imported malaria cases were predominantly among young adults, and all were male, with substantial majority were being either illiterate or self-educated. This is consistent with existing evidence that young men are the dominant group involved in seasonal labor migration to malaria-endemic areas in neighboring countries, often with little to no formal education [15–17]. Comparable trends and risks related to malaria importation have also been observed in other Southeast Asian neighboring countries [4,5]. Similar risks associated with labor migration and malaria importation have also been documented across the Greater Mekong Subregion, where young, mobile, and underserved male workers are particularly vulnerable [18]. This suggest that the demographic characteristics of imported malaria cases are closely tied to broader regional labor migration dynamics, which inherently shape exposure risk and disease transmission pathways.

All individuals had entered India through the ground crossing point at Kathkuiya which does not have a formal health desk. Nepal has established 20 designated Point of Entry (PoE) equipped with health desk offering services like disease screening, counseling, and health education for mobile and migrant populations [19]. However, many labor migrants such as those in this study tend to travel through informal or nearby crossings like Kathkuiya, which are not among the officially designated PoE. This is likely due to Nepal’s long and porous border with India, which allows for easy and unrestricted cross-border movement [20]. Similar patterns have been observed in other border regions of South Asia, where migrants often bypass official checkpoints due to convenience, familiarity, or the absence of border enforcement [21]**.** The fact that all participants used the same informal route highlights how predictable and localized cross-border movement can be, and suggests that mobility patterns are shaped more by community-level practices than by national-level infrastructure or policy while also illustrating both the potential value and inherent limitations of border-based interventions. These findings suggest that site specific and cohort focused engagement targeting well defined migrant communities may be a more efficient and realistic approach for addressing imported malaria in low-burden, last-mile settings such as Nepal [22].

In this study, the majority of imported malaria cases involved travel to Maharashtra, primarily to Mumbai. Specific destinations included Bhendi Bazar, Bohri Muhalla, and Null Bazaar. This finding aligns with reports showing that five administrative wards in South Mumbai—G South, E, F South, G North, and D—accounted for most of the city’s malaria cases. Notably, D Ward includes Bhendi Bazar, Bohri Muhalla, and Null Bazaar, the localities most frequently mentioned by the participants [23]. These areas are characterized by high population density, and congested housing conditions both of which are well-known contributors to mosquito breeding and to sustain malaria transmission in urban settings [24]. Surveillance data suggest that imported malaria cases are more often linked to large Indian cities where migrants go for work rather than to districts along the immediate border [25]. Previous research has shown that seasonal labor migration to states like Maharashtra, Gujarat, Assam, and West Bengal is a key driver of malaria importation into Nepal [20]. While India's national malaria burden has declined significantly in recent years [4], transmission persists in specific urban pockets [26], particularly in areas with high levels of construction, water storage, and poor vector control in informal settlements [27,28]. These findings highlights that the risk of malaria among Nepali labor migrants is not random, but concentrated in well-defined urban hotspots that maintain active transmission despite overall national progress. Travel to these destinations also appeared to be influenced by participants religion. In Narainapur municipality, imported malaria cases were concentrated among Muslim migrants, despite the community not being the majority population. This pattern suggests that labor migration is shaped by social and cultural networks linking Nepali Muslim workers with long-standing Muslim neighborhoods in South Mumbai [29]. Such networks offer familiarity, accommodation, and employment opportunities, but also anchor migrants in urban pockets where malaria transmission persists. This highlights how cultural ties and settlement patterns intersect with disease risk, shaping the demographic profile of malaria importation. Importantly, elimination strategies could be strengthened by engaging migrant networks and tailoring interventions to the social and occupational contexts that drive mobility and settlement.

Most of the migrants in this study worked in event-based catering services, involving food preparation, serving, dishwashing, and kitchen support during functions or gatherings. This work commonly requires both indoor and outdoor activity and is characterized by irregular and often late working hours. Previous research has shown that Nepali migrants frequently engage in evening and nighttime work (security guards) in malaria-endemic regions of India, including late-hour occupational activities, which increases their exposure to mosquito bites during peak vector biting hours [15]. A large proportion of participants lacked fixed housing and reported sleeping on the streets, partly due to late and irregular working hours. Many migrants reported traveling to malaria-endemic regions for short, repeated periods of approximately 2–3 months at a time, which reduced the feasibility of seeking rental accommodation. Furthermore, these workers often aimed to minimize living expenses in order to maximize savings, making rented housing less desirable during temporary employment, limiting their ability to consistently use bed nets or other standard protective measures. This may explain why all respondents preferred seasonal chemoprophylaxis over LLIN, which requires more stable sleeping arrangements. These conditions, night work, unstable shelter, and inconsistent protection collectively heighten exposure to malaria vectors and limit the effectiveness of conventional prevention strategies. Previous evidence has also linked prolonged exposure to mosquitoes due to work or living conditions with increased malaria transmission risk [30,31]. Together, these findings point to the importance of considering occupational and environmental factors when assessing malaria risk among migrant populations, as these factors directly shape both exposure and vulnerability.

In this study, the majority of imported malaria cases were caused by *P. vivax,* a species with distinct biological and programmatic implications for malaria elimination. Unlike other malaria species, *P. vivax* can form dormant liver stages and causes relapses weeks or even months after the initial infection, leading to repeated episodes of illness and ongoing health and economic burdens for affected individuals [32]. In near elimination settings such as in Nepal, relapses among returning migrant workers also increase the risk of local transmission if cases are not detected and treated in a timely manner. When combined with frequent cross border movement, the relapse biology of *P. vivax* may contribute to sustained transmission across borders, even where overall malaria incidence is low [33]. These features highlight the importance of strong surveillance systems, complete radical cure, and targeted follow up of imported *P. vivax* cases to safeguard progress toward malaria elimination.

### Strengths and limitations

#### Strengths.

This study focused on Narainapur, a high-burden municipality for imported malaria in Nepal, providing in-depth insights into a particularly vulnerable population. By collecting primary data through direct interviews with confirmed cases, it captured rich, context-specific information. Importantly, it identified clear geographic (e.g., South Mumbai neighborhoods) and occupational (e.g., catering services with night shifts) risk factors, offering actionable evidence for targeted malaria interventions. These findings directly support national elimination efforts by clarifying how labor migration patterns contribute to sustained malaria transmission.

#### Limitations.

This study was limited to individuals diagnosed within the health system in Narainapur, potentially excluding unreported or untreated cases and relied heavily on self-reported data. As the study was conducted over a one-year period, it provides only a cross-sectional snapshot and does not capture seasonal variations or long-term trends in imported malaria. Furthermore, since the study was restricted to a single municipality, the findings may not be generalizable to other regions where migrant occupations, travel patterns, and malaria exposure risks may vary significantly. Although efforts were made to reach all reported cases, some individuals particularly those currently abroad could not be contacted. This may have led to the underrepresentation of certain subgroups, such as younger or more mobile individuals, whose travel behaviors and risk profiles may differ. Future studies should consider strategies to capture data from this populations to ensure a more comprehensive understanding of imported malaria dynamics. This study did not capture month specific timing of stay at destination or its alignment with local malaria transmission seasons, which may have provided additional insight into seasonal exposure risk.

#### Conclusion.

This study shows that imported malaria in Narainapur continues to affect a clearly defined and vulnerable group, young male labor migrants, largely from the Muslim community, whose work and living conditions place them at ongoing risk of infection. The risk is not random but concentrated in well-defined urban hotspots in destination countries. Frequent travel, night and mixed indoor–outdoor work, and unstable housing make malaria exposure difficult to prevent using conventional control measures. As Nepal moves closer to malaria elimination, the continued occurrence of imported malaria within this group highlights how remaining malaria risk is increasingly concentrated among mobile and socially marginalized populations rather than the wider community.

#### Implications.

These findings highlight the need for malaria control strategies that better reflect the realities faced by migrant workers in Narainapur. Standard prevention tools, such as long-lasting insecticidal nets, are often difficult to use for individuals who travel frequently, work at night, or lack stable housing, underscoring the need for flexible and practical approaches tailored to migrants’ occupational and living conditions. Community engagement efforts should therefore prioritize male, Muslim, and highly mobile populations through culturally appropriate communication and trusted local networks, alongside strengthened pre-travel education, access to malaria chemoprophylaxis, and timely diagnosis and treatment. Incorporating migration and occupational information into routine surveillance systems could enable earlier identification of imported cases and more targeted responses, while extending health services to informal border crossings may strengthen border health security. Given the cross-border nature of labor migration and malaria exposure, closer collaboration with health authorities in destination areas is also essential to address shared transmission risks through coordinated surveillance, information sharing, and aligned prevention strategies, thereby supporting Nepal’s malaria elimination goals.

## Supporting information

S1 File Inclusivity in global research questionnaire. (DOCX)

S1 Text Questionnaire. (PDF)

S1 Data Deidentified analytic data. (XLSX)
