# Supplementary material for: Understanding imported malaria in western Nepal: Implications for malaria control and elimination
Source: PLOS Glob Public Health. 2026 Mar 27;6(3):e0006079. doi: 10.1371/journal.pgph.0006079 (PMC13029726; doi:10.1371/journal.pgph.0006079)
Supplement: S1 Text — (PDF) [file pgph.0006079.s002.pdf]

Title: Understanding Imported Malaria: Geographic Origins and Their Association with Plasmodium vivax Infection in Narainapur Municipality, Banke, Nepal, 2024

Name of interviewer: _________________

Date: ___________________

ID [_] [_] [_]

**Section 1: Socio-demographic and Socio-economic characteristics**		**Code**
SDC01	What is your age? [in years completed] ……….	SDC01 [_] [_]
SDC02	What is your Sex?<br>1. Male<br>2. Female	SDC02 [_]
SDC03	What is your Ethnicity?<br>1. Musalman 5. Janajati<br>2. Brahmin 6. Dalit<br>3. Newar 7. Others<br>4. Chhetri	SDC03 [_]
SDC04	What is your Religion?<br>1. Islam 4. Kiratism<br>2. Hinduism 5. Christianity<br>3. Buddhism 6. Others	SDC04 [_]
SDC05	What is your marital status?<br>1. Never married<br>2. Married<br>3. Divorced<br>4. Widowed /widower<br>5. Others	SDC05 [_]
SDC06	What is your ward number in Narainapur?	SDC06 [_]

	1. Ward 1 4. Ward 4<br>2. Ward 2 5. Ward 5<br>3. Ward 3 6. Ward 6	
SDC07	What is your education Status?<br>1. No education<br>2. Read and write (no formal education)<br>3. Primary (1-5 grades)<br>4. Secondary (6-9 grades)<br>5. SLC and higher secondary<br>6. University degree or higher	SDC07 [_]
SDC08	What is your approximate income per month?<br>1. ≤ 20,000<br>2. 21,000 - 40,000<br>3. 41,000 - 60,000<br>4. ≥ 60,000	SDC08 [_]
SDC08	SDC08_A Which country you travelled last and got malaria?<br>1. India<br>2. Other<br>SDC08_B<br>If other country, mention country Name<br>……………………………….	SDC08_A [_]<br><br><br><br><br>SDC08_B [_]
SDC09	Purpose of Travel<br>1. Work<br>2. Study<br>3. Visit friends and family<br>4. Tourist/Travel<br>Other……………….	SDC09 [_]
SDC10	Border Crossing point (Nepal)<br>…………………	SDC10 [_]

SDC11	Destination State (India)<br>…………………	SDC11 [_]
SDC12	Transit Point<br>House to……………….. (1st transit)<br>…………. To …………… (second transit)<br>………….. To …………….(third transit)<br>……………To……………….(destination)	SDC 12 [_]
SDC13	Total Transit Duration (total days to reach destination)<br>……………….. days	SDC13 [_]
SDC10	SDC10 Residence address at foreign land<br>SDC10_A State<br>SDC10_B City<br>SDC10_C Area<br>SDC10_D locality<br>SDC10_E Street	 SDC10_A [_]<br>SDC10_B [_]<br>SDC10_C [_]<br>SDC10_D [_]<br>SDC10_E [_]
SDC11	SDC11 Work address at foreign land<br>SDC11_A State<br>SDC11_B City<br>SDC11_C Area<br>SDC11_D locality<br>SDC11_E Street<br>SDC11_F Factory/Company Name	 SDC11_A [_]<br>SDC11_B [_]<br>SDC11_C [_]<br>SDC11_D [_]<br>SDC11_E [_]<br>SDC11_F [_]
SDC12	What was your Occupation?<br>1. Laborer<br>2. Security Guard/Watchman<br>3. Factory Worker<br>4. Hotel worker<br>5. Agriculture<br>6. Security Personnel (Police, Army etc)<br>7. Others………………..	SDC012 [_]

	Work outside or indoor<br>1. Outside always<br>2. Indoor always<br>3. Outside or Indoor	
SDC13	What was the usual timing for your job?<br>1. Day time<br>2. Night time<br>3. Day and night (mix)	SDC13 [_]
SDC14	In what type of house did you stay in foreign land<br>1. Kaccha House (lightweight materials like tin, wood, thatch, mud, or other natural materials)<br>2. Pakka House (brick, concrete, and stone)<br>3. Semi-Pakka House (mix of permanent materials (like concrete and brick) and temporary materials (like tin, wood, or thatch)<br>4. No House to stay, spend nights in street	SDC14 [_]
SDC15	Screens on windows to keep mosquitoes out?<br>1. Yes<br>2. No	SDC15 [_]
SDC16	How many people live in your room?<br>1. Only me<br>2. Two people<br>3. Three people<br>4. Four people<br>5. More than 4	SDC16 [_]
SDC17	Frequency of visit<br>1. Once a year or less<br>2. 2 to 3 times a year	SDC17 [_]

	3. 4 to 5 times a year<br>4. > 5 times a year	
SDC18	How many times have you contracted with malaria?<br>1. 1 time<br>2. 2 time<br>3. 3 time<br>4. 4 time<br>5. 5 or more	SDC18 [_]
SDC19	Will you go again in future?<br>1. Yes<br>2. No	SDC19 [_]
SDC20	If yes, where?<br>…………………………	SDC20[_]
**Section 2: Clinical characteristics**		
CL01	When you went to foreign land?<br>YYYY-MM-DD	CL01 [_]
CL02	When you returned back from foreign land?<br>YYYY-MM-DD	CL02 [_]
CL03	Symptom onset date<br>YYYY-MM-DD	CL03 [_]
CL04	Diagnosis date<br>YYYY-MM-DD	CL04 [_]
CL05	Plasmodium Species type<br>1. P. vivax<br>2. P. falciparum<br>3. P. mix<br>4. P. malaria<br>5. P. ovale<br>6. P. knowlesi	CL05 [_]

CL06	Signs and Symptoms (Multiple answers)<br>1. Fever<br>2. Chills and Rigor<br>3. Headache & Body ache<br>4. Nausea & Vomiting<br>5. Abdominal distension	CL06 [_]
CL07	CL07_A Admitted in hospital for malaria treatment?<br>1. Yes<br>2. No<br><br>CL07_B If Yes, how many Days …………..	CL07_A [_]<br><br>CL07_B [_]
CL08	How many days did you take anti-malarial medications? (select nearest answer)<br>1. 3 days<br>2. 7 days<br>3. 14 days<br>4. Don't remember	CL08 [_]
**Section 3: Behavioral Characteristics**		
BC01	Is malaria a preventable disease?<br>1. Yes<br>2. No<br>3. Don't know	BC01 [_]
BC02	Did you receive malaria related information before travel?<br>1. Yes<br>2. No	BC02 [_]
BC03	What measures do you usually take to prevent malaria? (multiple answers)<br>1. Sleeping under bed nets	BC03 [_]

	2. Wearing long sleeves and pants during peak mosquito activity time<br>3. Mosquito repellents use<br>4. Mosquito spray/coil/liquid	
BC03	BC03_A If you were given chemoprophylaxis for free before traveling (one tablet weekly, starting two weeks prior to travel, during your stay, and for four weeks after returning to Nepal), would you consume it regularly?<br>1. Yes<br>2. No<br><br>BC03_B If No what is the reason<br>1. Fears of side effects<br>2. Cultural Beliefs<br>3. Doubt About Effectiveness<br>4. It's unnecessary (Low Perceived Risk)<br>5. Stay longer duration inconvenient to follow<br>6. Pre-existing health issues<br>7. others	BC03_A [_]<br><br>BC03_B [_]
BC04	BC04_A If you were given Long Lasting Insecticidal Nets (LLIN) for free before traveling, would you use it regularly?<br>1. Yes<br>2. No<br><br>BC04_B If No what is the reason<br>1. Lack of Awareness<br>2. Inconvenience in setting up or using the nets properly	BC04_A [_]<br><br>BC04_B [_]

	3. Cultural Practices<br>4. Concerns about heat or discomfort while sleeping under a net<br>5. Low Perceived Risk<br>6. Reliance on other methods of prevention<br>7. Others	
